# Non-volatile signals and redox mechanisms are required for the responses of Arabidopsis roots to *Pseudomonas oryzihabitans*

**DOI:** 10.1093/jxb/erac346

**Published:** 2022-08-24

**Authors:** Daniel Cantabella, Barbara Karpinska, Neus Teixidó, Ramon Dolcet-Sanjuan, Christine H Foyer

**Affiliations:** Institute of Research and Agrofood technology (IRTA) Postharvest Program, Edifici Fruitcentre, Parc Científic i Tecnològic Agroalimentari de Lleida, 25003 Lleida, Catalonia, Spain; IRTA, Plant In Vitro Culture Laboratory, Fruticulture Program, Barcelona, Spain; School of Biosciences, University of Birmingham, Edgbaston, Birmingham B15 2TT, UK; School of Biosciences, University of Birmingham, Edgbaston, Birmingham B15 2TT, UK; Institute of Research and Agrofood technology (IRTA) Postharvest Program, Edifici Fruitcentre, Parc Científic i Tecnològic Agroalimentari de Lleida, 25003 Lleida, Catalonia, Spain; IRTA, Plant In Vitro Culture Laboratory, Fruticulture Program, Barcelona, Spain; School of Biosciences, University of Birmingham, Edgbaston, Birmingham B15 2TT, UK; Nanjing Agricultural University, China

**Keywords:** Ascorbate, ethylene-responsive transcription factor 109, glutathione, plant growth-promoting rhizobacteria, *Pseudomonas oryzihabitans*, reactive oxygen species, root system architecture

## Abstract

Soil bacteria promote plant growth and protect against environmental stresses, but the mechanisms involved remain poorly characterized, particularly when there is no direct contact between the roots and bacteria. Here, we explored the effects of *Pseudomonas oryzihabitans* PGP01 on the root system architecture (RSA) in *Arabidopsis thaliana* seedlings. Significant increases in lateral root (LR) density were observed when seedlings were grown in the presence of *P. oryzihabitans*, as well as an increased abundance of transcripts associated with altered nutrient transport and phytohormone responses. However, no bacterial transcripts were detected on the root samples by RNAseq analysis, demonstrating that the bacteria do not colonize the roots. Separating the agar containing bacteria from the seedlings prevented the bacteria-induced changes in RSA. Bacteria-induced changes in RSA were absent from mutants defective in ethylene response factor (ERF109), glutathione synthesis (*pad2-1*, *cad2-1*, and *rax1-1*) and in strigolactone synthesis (*max3-9* and *max4-1*) or signalling (*max2-3*). However, the *P. oryzihabitans-*induced changes in RSA were similar in the low ascorbate mutants (*vtc2-1*and *vtc2-2*) to the wild-type controls. Taken together, these results demonstrate the importance of non-volatile signals and redox mechanisms in the root architecture regulation that occurs following long-distance perception of *P. oryzihabitans*.

## Teaser

Perception of the plant growth-promoting rhizobacterium *Pseudomonas oryzihabitans*modifies the Arabidopsis root transcriptome and root system architecture through phytohormone- and redox-dependent pathways.

## Introduction

Plants live in harmony with soil microbiome communities, with whom they are in constant chemical communication. Soil bacteria and fungi can influence plant growth and performance, particularly through effects exerted at the seedling stage (Zhang *et al.*, 2022). Plant growth-promoting rhizobacteria (PGPR) are comprised of different orders of bacterial species. They not only modulate plant growth and root system architecture (RSA) but they also trigger host immune responses (Poitout *et al.*, 2017; Shekhar *et al.*, 2019). Soil-borne plant pathogens can be controlled by the status of the soil microbiome, in what is known as ‘disease-suppressive soil effects’, which rely heavily on competition for plant nutrients between the different microorganisms (Schlatter *et al.*, 2017). PGPR also produce compounds such as cyclic lipopeptides, polyketides, and bacteriocins that can have a direct negative effect on soil pathogens (Andric *et al.*, 2021).

PGPR modulate RSA by regulating the production of phytohormones such as gibberellic acid (GA), auxin [indole acetic acid (IAA)], abscisic acid, and salicylic acid (SA) (Yuhashi *et al.*, 2000; Poitout *et al.*, 2017; Niu *et al.*, 2018). Some PGPR species such as *Pseudomonas aeruginosa*, *Klebsiella* spp., *Rhizobium* spp., and *Mesorhizobium* spp. secrete IAA and so directly regulate RSA (Ahemad and Kibret, 2014). Such mutualistic interactions enhance the capacity of roots to take up nutrients (Glick, 2012). PGPR also improve the solubilization of minerals such as phosphorus, zinc, and potassium, and increase iron sequestration by siderophore production. Several *Rhizobium* species secrete nitrogenases that improve the fixation of nitrogen in anaerobic soils, as well as releasing organic acids to increase phosphorus uptake (Yanni *et al.* 2001).

The control of lateral root (LR) development involves a network of phytohormones that includes auxin and strigolactones (SLs; Sharma *et al.*, 2020). SLs inhibit branching (Kapulnik *et al.*, 2011; Rasmussen *et al.*, 2012) and interact with other phytohormones, particularly auxins, to control overall root morphology (Agusti *et al.*, 2011; Ruyter-Spira *et al.*, 2011; De Jong *et al.*, 2014). SLs also participate in the regulation of plant stress responses (Foo and Reid, 2013; De Jong *et al.*, 2014; Quain *et al.*, 2014; Cooper *et al.*, 2018). Crucially, they are important regulators of plant–microbe interactions. For example, the SLs present in root exudates attract arbuscular mycorrhizal fungi and they also stimulate the nodulation process in legumes (López-Ráez *et al.*, 2017).

Reactive oxygen species (ROS) are important components of the phytohormone signalling pathways that control RSA (Manzano *et al.*, 2014; Kong *et al.*, 2018; Yamada *et al.*, 2020; Eljebbawi *et al.*, 2021). For example, the control of ROS accumulation is an important factor in the emergence of LR primordia and it also influences the number of pre-branch sites (Orman-Ligeza *et al.*, 2016). Transcription factors such as ethylene response factor (ERF)109 (also called redox-responsive transcription factor 1) are crucial regulators of the responses of RSA to environmental cues through modulation of jasmonate (JA), ethylene, and ROS signalling (Cai *et al.*, 2014; Matsuo *et al.*, 2015). While the effects of PGPR on plant morphology have been extensively studied, little attention has as yet been paid to the roles of ROS and redox signalling in plant–bacteria interactions, particularly when there is no direct contact between the roots and bacteria.

The non-fermenting yellow-pigmented, Gram-negative, lactose- and oxidase-negative rod-shaped bacterium, *Pseudomonas oryzihabitans* PGP01 (also known as *Chromobacterium typhiflavum* and *Flavimonas oryzihabitans*), is an opportunistic human pathogen. This saprophytic bacterium has been isolated from a range of human wound and soft tissue infections, leading to septicaemia, prosthetic valve endocarditis, and peritonitis. It also lives freely in soils as well as on medical and other equipment (Keikha *et al.*, 2019). In plants, *P. oryzihabitans* has been linked to panicle blight in rice (Hou *et al.*, 2020) and to stem and leaf rot in muskmelon (Li *et al.*, 2021). However, other studies have shown that *P. oryzihabitans* PGP01 can exert a positive effect on root growth (Belimov *et al.*, 2015; Cantabella *et al.*, 2020). The aims of the present study were firstly to determine the effects of *P. oryzihabitans* on RSA in *A. thaliana*, secondly to characterize how perception of *P. oryzihabitans* alters the root transcriptome profile, and thirdly to determine whether ROS-related mechanisms were involved in the responses of RSA to perception of the presence of the bacterium.

## Materials and methods

### Plant material and growth conditions

Seeds of the *A. thaliana* Columbia-0 (Col-0) wild-type (WT), the SL-deficient mutants (*max2-3*, *max3-9*, and *max4-1*), the ascorbate-deficient (*vtc2-1* and *vtc2-2*) mutants, the glutathione (GSH)-deficient (*pad2-1, cad2-1*, *and rax1-1*) mutants, a transformed line overexpressing ERF109 (*ov32*), and a mutant line lacking a functional transcription factor (*erf109*) were surface sterilized with 50% ethanol during 5 min, followed by three rinses with sterile distilled water. Sterile seeds were cultured on 10 cm square Petri dishes containing half-strength Murashige and Skoog medium (1/2 MS, pH 5.7), supplemented with 0.01% myo-inositol, 0.05% MES, 1% sucrose, and 1% plant agar. Plates were stored at 4 °C in a dark room for 2–4 d to synchronize germination. Seedlings were grown vertically in a controlled-environment cabinet at 22 °C with a 16 h photoperiod for 6 d.

### Inoculation of bacteria onto plates containing Arabidopsis seedlings

The growth-promoting bacterium *P. oryzihabitans* strain PGP01 was obtained from the IRTA Postharvest Plant Growth Promoter Microorganism (PGPM) Collection (Lleida, Catalonia, Spain). Bacteria were grown in nutrient yeast dextrose agar (NYDA: nutrient broth, 8 g l^–1^; yeast extract, 5 g l^–1^; dextrose, 10 g l^–1^; and agar, 20 g l^–1^) media for 48 h. Bacteria were applied to plates containing 6-day-old Arabidopsis seedlings according to the method of Zamioudis *et al.* (2013). Bacteria were collected in 10 mM MgSO_4_, and washed by centrifugation at 5000 *g* for 5 min. After resuspension in 10 mM MgSO_4_, the bacterial concentration was adjusted to 1 × 10^6^ by measuring turbidity at 600 nm. Aliquots (50 µl) of bacteria were applied at a distance of 5 cm from the root tip of 6-day-old Arabidopsis Col-0 seedlings. A concentration of 1 × 10^6^ colony-forming units (CFU) ml^–1^ was used to examine the effects of the presence of bacteria on root architecture.

For the experiments designed to determine whether volatile signals were involved in root responses to *P. oryzihabitans*, 1 cm sections of the agar were removed from plates so as to physically separate the agar containing seedlings from the agar containing bacteria, as illustrated in Supplementary Fig. S1.

### Measurements of root architecture

After 7 d of co-culture with bacteria, pictures of control and bacteria-treated plates were taken, and different parameters such as primary root (PR) length, number of visible LRs, and length of LRs were measured using ImageJ software. LR density was calculated by dividing the number of LRs by the PR length for each root analysed, as described previously (Dubrovsky and Forde, 2012). The LR density method provides a measure of the number of LRs per unit length of PR and allows a comparison of LR formation in PRs with different elongation rates.

### RNAseq analysis

The roots of Arabidopsis seedlings were harvested after 7 d growth in the absence or presence of bacteria and immediately frozen in liquid nitrogen. Each biological replicate contained roots from at least three plates, each of them with six seedlings. RNA was extracted from frozen root samples using TRIreagent® (SigmaAldrich). RNA quality was checked by Nanodrop, and RNA integrity was confirmed using a 0.8% agarose gel. RNAseq data were analysed as described previously (De Simone *et al.*, 2017).

### Statistical analysis

All of the experiments were repeated at least three times. Data represent the mean ±SE of the mean. Data from the experiments using Col-0 and bacteria were analysed by one-way ANOVA and also by a pairwise *t*-test. A two-way ANOVA was also performed on the data from studies on SL, ascorbate, and GSH mutants. Statistical significance was judged at the level *P*<0.05, and Duncan’s post-hoc test was used for the means separation when the differences were significant using the IBM SPSS statistics 25 program.

## Results

**Fig. 1.**
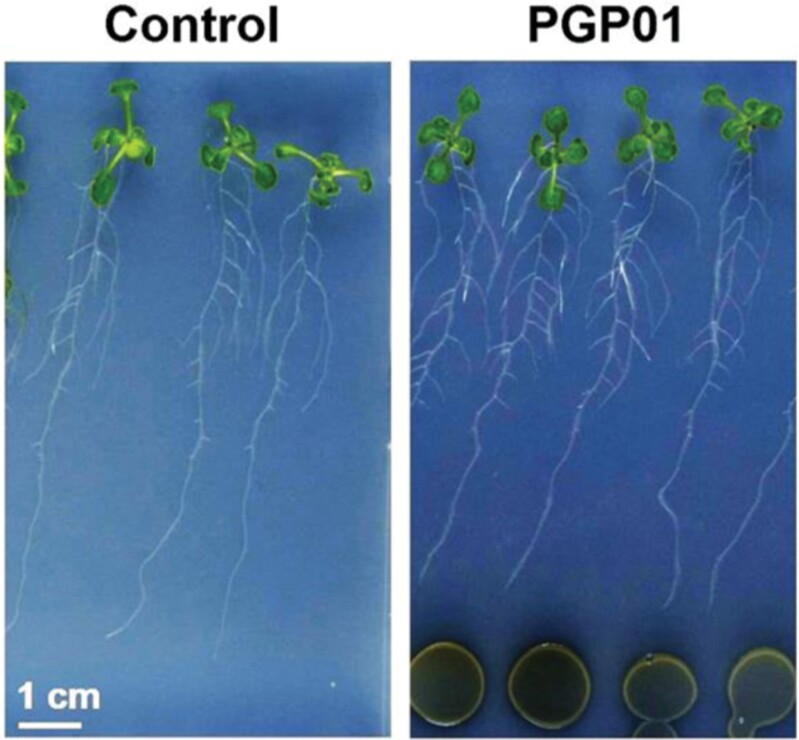
Representative images of wild-type Arabidopsis seedlings that had been grown for 6 d in the absence of *P. oryzihabitans* and then for a further 7 d in either the absence (control) or the presence of bacteria (PGP01).

**Fig. 2.**
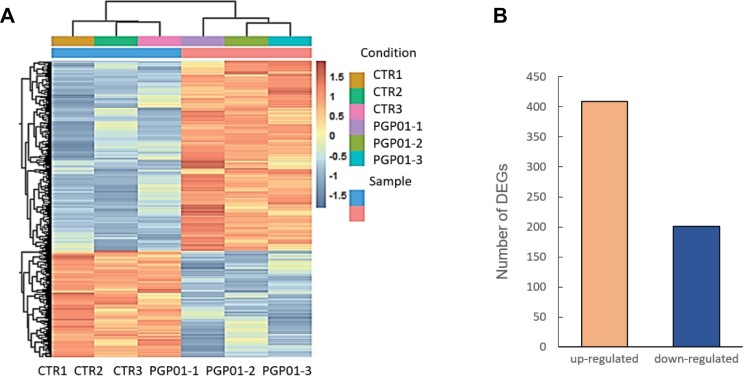
Differentially expressed transcripts in the roots of the wild type (A) and number of transcripts significantly increased and decreased (B). Seedlings had been grown for 6 d in the absence of *P. oryzihabitans* and then for a further 7 d in either the absence or presence of bacteria.

Previous studies have shown that the presence of *P. oryzihabitans* PGP01 induces modifications in *Pyrus* and *Prunus* rootstocks (Cantabella *et al.*, 2020, 2021). The data presented in Fig. 1 demonstrate that perception of *P. oryzihabitans* PGP01 also induces changes in RSA in Arabidopsis. In these studies, *P. oryzihabitans* was placed on the same plates but not touching the roots of the Arabidopsis seedlings (Fig. 1). Transcriptome profile comparisons of the roots of seedlings grown on plates in the absence or presence of bacteria were measured 7 d after plating (Fig. 2A; Supplementary Table S1); The RNAseq analysis revealed the absence of bacterial transcripts from the roots of Arabidopsis plants (Supplementary Table S1). In total, 409 transcripts were increased in abundance in the roots grown in the presence of *P. oryzihabitans* compared with those grown in the absence of bacteria, and 201 transcripts were less abundant (Fig. 2B).

### Root transcriptome responses to bacteria

**Fig. 3.**
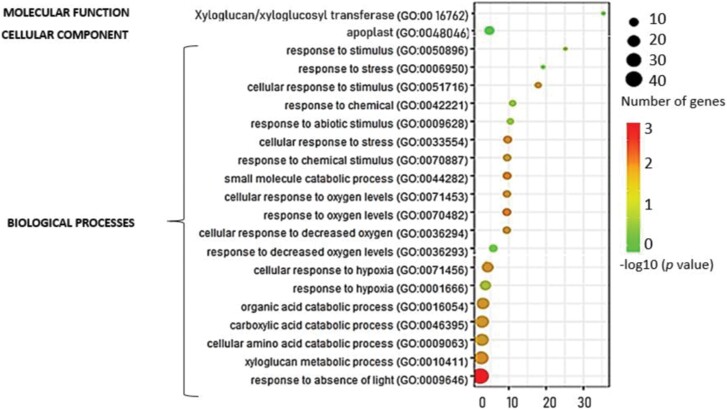
Gene Ontology (GO) analysis showing the biological processes involved in root responses to *P. oryzihabitans*.

A functional analysis of differentially expressed genes (DEGs) in response to *P. oryzihabitans* PGP01 (Fig. 3A) reveals Gene Ontology (GO) terms included are response to absence of light (GO:0009646), xyloglucan metabolism (GO:0010411), cellular amino acid metabolism (GO:00009063), carboxylic acid catabolism (GO:0046395), organic acid catabolism (GO:0016054), and several terms related to hypoxia and decreased oxygen availability (GO:0036294, GO:0070482, GO:0001666, GO:00771456, GO:0036293, and GO:0071453). Other terms such as cellular response to chemical stimulus (GO:0051716, GO:00770887, GO:0042221, and GO:0050896) and response to abiotic stress (GO:0033554, GO:0009628, and GO:0006950) were present, as were terms related to the apoplast (GO:0048046) and xyloglucan/xylotransferase activity (GO:0016762).

**Fig. 4.**
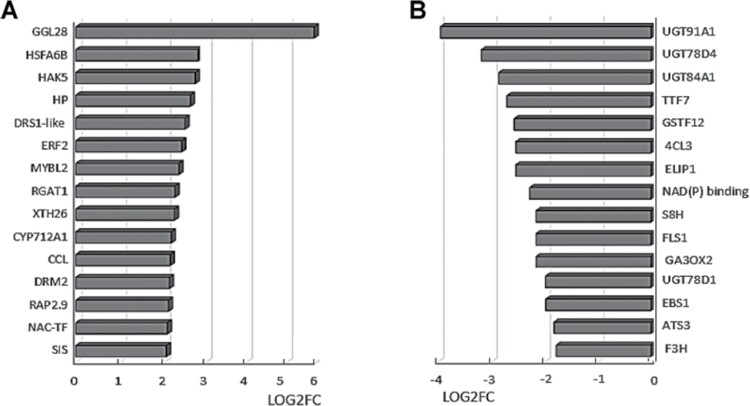
Transcripts that were most increased (A) or decreased in abundance (B) in response to the presence of *P. oryzihabitans* PGP01. (A) AT5G45950 (GGL28, GDSL-motif esterase/acetyltransferase/lipase); AT3G22830 (HSFA6B, heat stress transcription factor A-6b), AT4G13420 (HAK5, potassium channel); AT2G39980 (HP, hypotetical unknown protein); AT5G28610 (DRS1-like, ATP-dependent RNA helicase); AT5G47220 (ERF2, ethylene response factor 2); AT1G71030 (MYBL2, myb family transcription factor); AT1G19530 (RGAT1, RGA target 1); AT4G28850 (XTH26, xyloglucan endotransglucosylase 26); AT2G42250 (CYP12A1, cytochrome P450); AT3G26740 (CCL, circadian control of mRNA stability); AT2G33830 (DRM2, dormancy/auxin associated protein 2); AT4G06746 (RAP2.9, ERF/AP2 transcription factor family); AT1G69490 (NAC-TF, transcription factor); AT5G02020 (SIS, salt-induced serine rich). (B) AT2G22590 (UGT91A1, UDP-glucosyltransferase 91A1); AT5G17040 (UGT78D4, UDP-glucosyltransferase 78D4); AT4G15480 (UGT84A1, UDP-glucosyltransferase 84A19); AT5G07990 (TT7, flavonoid 3ʹ hydroxylase activity); AT5G17220 (GSTF12, glutathione *S*-transferase 12); AT1G65060 (4CL, 4-coumarate:CoA ligase); AT3G22840 (ELIP1, early light inducible 1); AT2G23910 [NAD(P) binding, Rossmann-fold superfamily]; AT3G12900 (S8H, scopoletin 8 hydrolase); AT5G08640 (FLS1, flavonol synthase 1); AT1G80340 (GA3OX2, gibberellin 3-oxidase 2); AT1G30530 (UGT78D1, UDP-glucosyl transferase 78D1); AT4G17680 (EBS1, exclusivly sensitive to bicarbonate 1); AT5G62210 (ATS3, embryo-specific protein 3); AT3G51240 (F3H, flavanone 3-hydroxylase).

The genes that were highly expressed in response to *P. oryzihabitans* (Fig. 4A) include those encoding a guard cell-enriched lipase called GGL28 (GDSL-like), heat shock factor (HSF) A6b, high affinity K^+^ transporter HAK5, and the MYB transcription factor MYBL2 (Fig. 2A). Transcripts encoding ethylene response factor 2 (ERF2), ANACO29, and the related ERF/AP2 transcription factor family protein (RAP2.9) were also increased in roots exposed to *P. oryzihabitans* (Fig. 4A).

A small number of transcripts were decreased in abundance in response to *P. oryzihabitans* (Fig. 4B). These include mRNAs encoding UDP-glycosyltransferases (UGT91A1, UGT78D4, UGT84A1, and UGT78D1), as well as transcripts encoding transparent testa (TT) 7, glutathione *S*-transferase (GST) 26, and gibberellin 3-β-dioxygenase (GA3OX2; Fig. 4B).

**Fig. 5.**
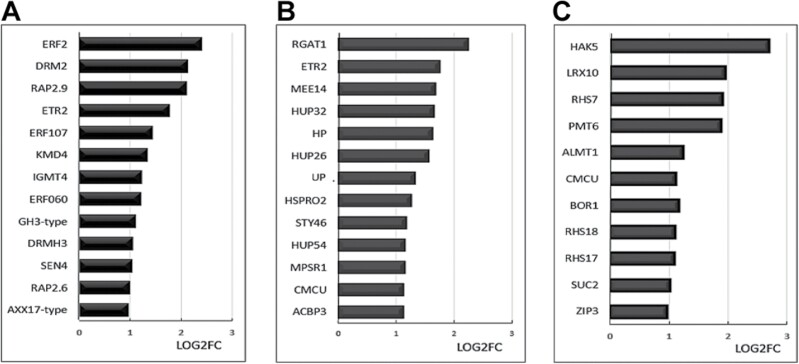
Subsets of transcripts involved in (A) phytohormone signalling, (B) hypoxia, and (C) nutrient status that were increased in abundance in the presence of *P. oryzihabitans* PGP01. (A) Responses to hormones: AT5G47220 (ERF2, ethylene-responsive transcription factor 2); AT2G33830 (DRM2, dormancy/auxin associated protein 2); AT4G06746 (RAP2.9, ethylene responsive RAP2.9); AT3G23150 (ETR2, ethylene response 2); AT5G61590 (ETR107, ethylene responsive transcription factor 107); AT3G59940 (KMD4, kiss me deadly 4, controls cytokinin signalling); AT1G21130 (IGMT4, indole glucosinolate-*O*-methyltransferase 4); AT4G39780 (ERF060, ethylene responsive factor 1); AT1G48690 (GH3-type, auxin responsive GH3-type protein); AT1G56220 (DRMH3, dormancy-associated protein homologue 3), AT4G30270 (SEN4, senescence 4, brassinosteroid response); AT1G43160 (RAP2.6, ethylene responsive factor RAP2.6). (B) Responses to hypoxia: AT1G19530 (RGAT1, RGA Target 1); AT3G23150 (ETR2; ethylene response 2); AT2G15890 (MEE14, maternal effect embryo arrest 14); AT1G33055 (HUP32, hypoxia response protein 32); AT5G65207 (HP, hypothetical protein responsive to hypoxia); AT3G10020 (HUP26, hypoxia response protein 26); AT1G10140 (UP, uncharacterized protein responsive to hypoxia); AT2G40000 (HSPRO2, orthologue sugar beet HSPRO2); AT4G38470 (STY46, serine/threonine kinase); AT4G27450 (HUP54, hypoxia response protein 54); AT1G26800 (MPSR1, misfolded protein sensing ring E3 ligase); AT5G66650 (CMCU, chloroplast-localized mitochondrial calcium uniporter); AT4G24230 (ACBP3, acyl-CoA-binding domain 3). (C) Transport facilitation and root growth: AT4G13420 (HAK5, potassium channel transporter 5); AT1G54970 (RHS7, root hair specific 7, ethylene regulated); AT4G36670 (PMT6, POLYOL/monosaccharide transporter 6); AT5G17860 (CCX4, cation/calcium exchanger); AT1G08430 (ALMT1, aluminium activated malate transporter); AT5G66650 (CMCU, chloroplast-localized mitochondrial calcium uniporter 3); AT2G47160 (BOR1, boron transporter 1); AT5G22410 (RHS18, root hair specific 18); AT4G38390 (RHS17, root hair specific 17); AT1G22710 (SUC2, sucrose protein symporter 2); AT2G32270 (ZIP3, zinc transporter 3 precursor).

Further analysis of the most enriched GO terms revealed that transcripts encoding some hormone-related proteins were more expressed in roots exposed to *P. oryzihabitans* (Fig. 5C). These include ERF2, ERF107, DORMANCY/AUXIN ASSOCIATED FAMILY PROTEIN 2 (DRM2), and KISS ME DEADLY 4 (KMD4) (Fig. 5A). Several transcripts associated with hypoxia responses (Fig. 5B) and nutrient acquisition and transport (Fig. 5C) were also increased in roots exposed to *P. oryzihabitans*.

### Root responses to bacteria in lines with modified expression of ERF109

**Fig. 6.**
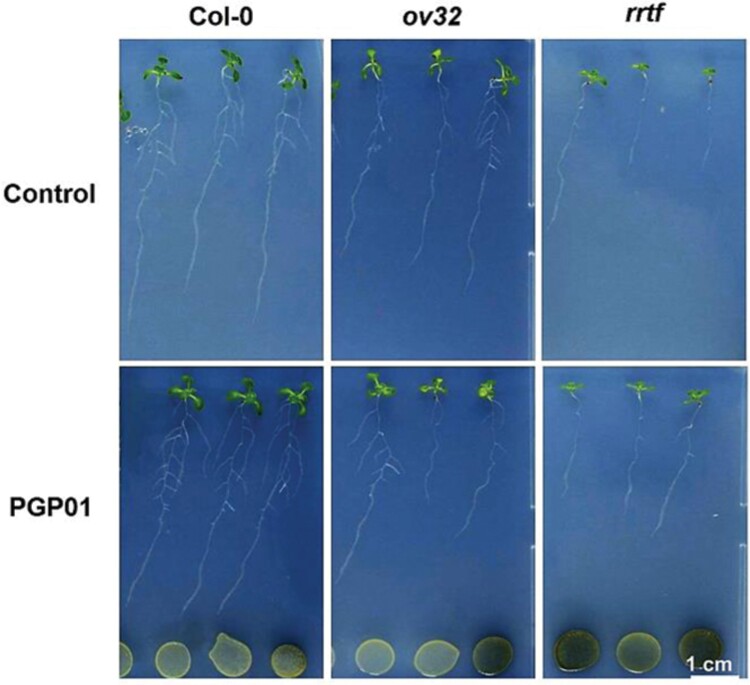
Representative images of wild-type Arabidopsis seedlings, seedlings overexpressing ERF109 (*ov32*), and *erf109* mutants. Seedlings had been grown for 6 d in the absence of *P. oryzihabitans* and then for a further 7 d in either the absence (control) or the presence of bacteria (PGP01).

**Fig. 7.**
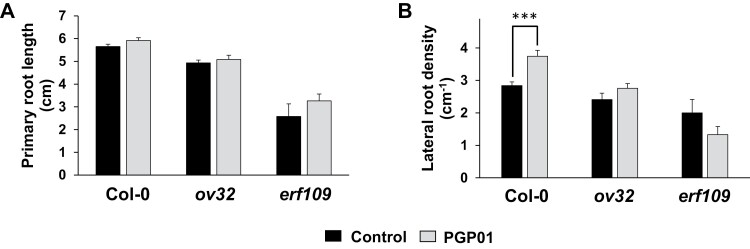
The effect of the presence of *P. oryzihabitans* PGP01 on primary root length (A) and lateral root density (B) in wild-type *A. thaliana*, a transgenic line overexpressing redox-responsive transcription factor 1 (*ov32*), and a *erf109* mutant line. Samples of bacterial inoculum was placed 5 cm away for the tips of the primary roots of 6-day-old seedlings that had been grown on agar plates. Root parameters were measured 7 d after inoculation. Data show the mean ±SE of three independent biological samples. Asterisks indicate significant differences according to *t*-test (*P*<0.05).

To analyse the role of ERF109 in root responses to *P. oryzihabitans*, RSA was compared in WT Arabidopsis seedlings, a transformed line overexpressing ERF109 (*ov32*), and a mutant line lacking a functional transcription factor (*erf109*; Fig. 6). The presence of bacteria increased LR density only in the WT (Fig. 7). LR density was not changed by perception of the bacteria in the *ov32* plants or the *erf109* mutants (Fig. 7).

### Root responses to bacteria in ascorbate-deficient mutants

**Fig. 8.**
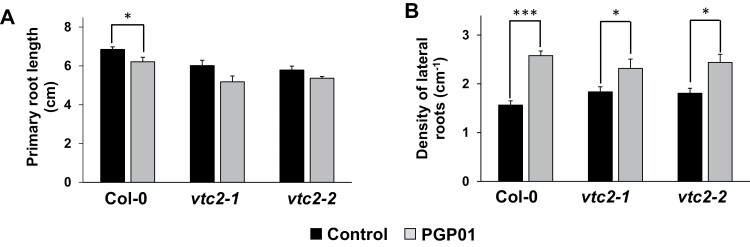
The effect of the presence of *P. oryzihabitans* PGP01 on primary root length (A) and lateral root density (B) in wild-type *A. thaliana* and mutants that are defective in ascorbate (*vtc2-1* and *vtc2-2*). Samples of bacterial inoculum were placed 5 cm away for the tips of the primary roots of 6-day-old seedlings that had been grown on agar plates. Root parameters were measured 7 d after inoculation. Data show the mean ±SE of three independent biological samples. Asterisks indicate significant differences according to *t*-test (*P*<0.05).

Two independent lines of ascorbate-deficient, vitamin C (*vtc2*) mutants were used to analyse the role of this low molecular weight antioxidant buffer in root responses to *P. oryzihabitans* (Fig. 8). LR densities were similar in all genotypes in the absence of bacteria (Fig. 8B). Moreover, the presence of *P. oryzihabitans* significantly increased LR density in all genotypes (Fig. 8).

### Root responses to bacteria in glutathione-deficient mutants

**Fig. 9.**
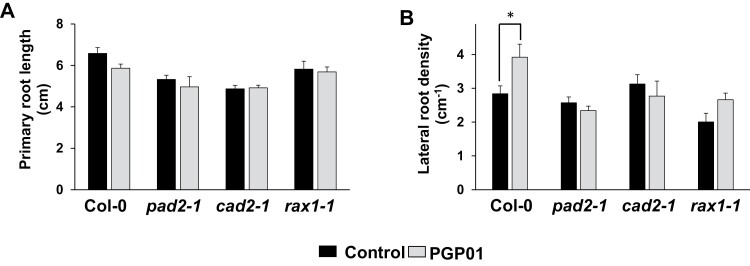
The effect of the presence of *P. oryzihabitans* PGP01 on primary root length (A) and lateral root density (B) in wild-type *A. thaliana* and mutants that are defective in glutathione (*cad2-1*, *pad2-1*, and *rax1-1*). Samples of bacterial inoculum were placed 5 cm away for the tips of the primary roots of 6-day-old seedlings that had been grown on agar plates. Root parameters were measured 7 d after inoculation. Data show the mean ±SE of three independent biological samples. Asterisks indicate significant differences according to *t*-test (*P*<0.05).

Three independent lines of GSH-deficient mutants [*phytoalexin-deficient 2* (*pad2-1*), the *cadmium-sensitive* 2 (*cad2-1*), and the *regulator of APX2-1* (*rax1-1*)], which accumulate less glutathione (~30%) than the WT (Schnaubelt *et al.*, 2015) were used to analyse the role of the low molecular weight antioxidant in root responses to *P. oryzihabitans.* The PRs of all genotypes were not significantly changed by the presence of *P. oryzihabitans* (Fig. 9A). Moreover, the presence of *P. oryzihabitans* significantly increased LR density in the WT roots but not in those of the *cad2-1*, *pad2-1*, and *rax1-1* mutants (Fig. 9B).

### Root responses to bacteria in SL-deficient mutants

**Fig. 10.**
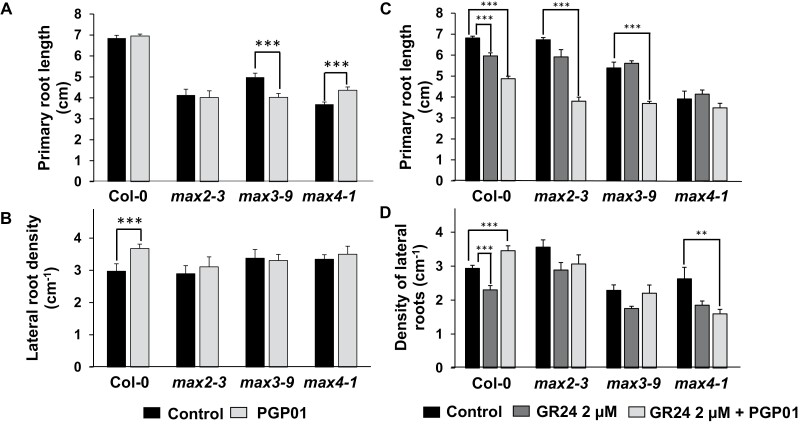
The effect of the presence of *P. oryzihabitans* PGP01 on primary root length (A) and lateral root density (B) in wild-type *A. thaliana* and mutants that are defective in SL synthesis (*max3-9* and *max4-1*) and signalling (*max2-3*). Samples of bacterial inoculum were placed 5 cm away for the tips of the primary roots of 6-day-old seedlings that had been grown on agar plates. Root parameters were measured 7 d after inoculation. Data show the mean ±SE of three independent biological samples. Asterisks indicate significant differences according to *t*-test (*P*<0.05).

The presence of bacteria increased LR density only in the WT. LR density was not changed by perception of the bacteria in mutants defective in SL synthesis or SL signalling (Fig. 10B). LR density was decreased in the WT in the presence of the synthetic SL GR24 but increased in the presence of GR24 and bacteria (Fig. 10D). In contrast, LR density was not significantly increased in the presence of GR24 and bacteria in any of the SL mutant lines (Fig. 10D). Moreover, bacteria-induced decreases in LR density were observed in the presence of GR24 in the roots of the *max 4-1* mutants (Fig. 10D).

### Root system architecture responses to *P. oryzihabitans* do not appear to be triggered by volatile signals

**Fig. 11.**
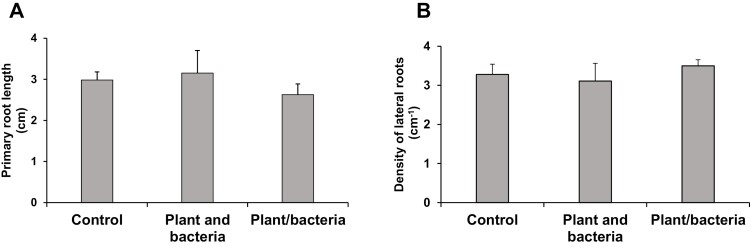
The effect of removal of the agar between *P. oryzihabitans* PGP01 and Arabidopsis seedings. Arabidopsis seedlings were either separated by a 1 cm gap in the agar (Control), separated by a 1 cm gap from seedlings grown in the presence of *P. oryzihabitans* (Plants and PGP01), or separated from agar on which *P. oryzihabitans* was grown (Plants/PGP01). Seedlings were grown for 6 d in the absence of *P. oryzihabitans* and then for a further 7 d in either the absence or presence of bacteria. Primary root length (A) and lateral root density (B).

To test whether volatile signals were involved in the interactions between *P. oryzihabitans* and Arabidopsis roots, 1 cm sections of the agar were removed from the plates. Thus, the agar containing seedlings was physically separated from the agar containing bacteria (Supplementary Fig. S1). PR lengths (Fig. 11A) and LR densities (Fig. 11B) were similar in seedlings separated by a 1 cm gap in the agar (Control), separated from seedlings grown in the presence of *P. oryzihabitans* (Plants and bacteria), or separated from agar on which *P. oryzihabitans* was grown (Plants/bacteria).

## Discussion

RSA undergoes fine tuning in response to cues from the soil microbiome (Hodge *et al.*, 2009; Ruiz Herrera *et al.*, 2015). For example, the presence of PGPR modifies RSA and primes plant defences against pathogens and herbivores through induced systemic resistance responses (Pieterse *et al.*, 2014; Rashid *et al.*, 2017; Veselova *et al.*, 2019). The data presented here demonstrate that remodelling of the root transcriptome and RSA occurs upon perception of *P. oryzihabitans*, without direct contact between the bacteria and the roots. However, root cap-derived signals from the soil microbiome were found to be important in the regulation of RSA (Crombez *et al.*, 2020). The root responses to *P. oryzihabitans* reported here involve subtle transcriptome remodelling and require SLs and redox signalling through GSH and ERF109, but not ascorbate. The RSA response was lost once the agar containing the seedlings was physically separated from that containing the bacteria, suggesting that volatile signals are not important drivers of root remodelling.

Considerable genetic variation in the ability of Arabidopsis accessions to benefit from root associations with *P. simiae* has been reported (Wintermans *et al.*, 2016). *Pseudomonas* species deploy a range of signals that modulate root development, including the secretion of phytohormones such as IAA and other small molecules, and the release of volatile organic compounds (VOCs; Zamioudis *et al.*, 2013). For example, *P. fluorescens* SS101 promotes plant growth through the release of 13-tetradecadien-1-ol, 2-butanone, and 2-methyl-*n*-1-tridecene (Park *et al.*, 2015) while *P. putida* and *P. fluorescens* produce cyclodipeptides such as cyclo(l-Pro-l-Val), cyclo(l-Pro-l-Phe), and cyclo(l-Pro-l-Tyr), which modulate the expression of auxin-responsive genes in roots (Ortiz-Castro *et al.*, 2020). *Pseudomonas oryzihabitans* PGP01 is able to produce IAA, when supplied with appropriate substrates (Cantabella *et al.*, 2021). Like other *Pseudomonas* strains, *P. oryzihabitans* PGP01 triggers auxin-dependent root developmental programmes including abundant LR formation (Ortiz-Castro *et al.*, 2011, 2020; Zamioudis *et al.*, 2013). The data presented here suggest that non-volatile signals are essential for the control root responses to *P. oryzihabitans* PGP01.

While volatile signals do not appear to be important in the control of RSA by *P. oryzihabitans* PGP01, the transcriptome signature reveals a role for ethylene signalling, which regulates auxin transport and the frequency of LR formation (Xu *et al.*, 2020). Transcripts encoding ERF2 and the related ERF/AP2 transcription factor family protein (RAP2.9) were more abundant in roots exposed to *P. oryzihabitans*. These transcription factors play crucial roles in immunity, regulating multiple SA, JA, and ROS signalling pathways (Yang *et al.*, 2021). Ethylene also stimulates the expression of senescence-associated genes such as *ANACO29* (Kim *et al.*, 2015), which is also highly expressed in roots exposed to *P. oryzihabitans*. Ethylene promotes the homeostasis of Na^+^/K^+^, nutrients, and ROS to enhance plant tolerance to salinity (Tao *et al.*, 2015).

The perception of *P. oryzihabitans* caused changes to the root transcriptome even though there was no direct colonization or physical contact between the organisms except through the agar. The genes that were most highly expressed in response to *P. oryzihabitans* include mRNAs encoding GDSL28 and HSFA6b. HSFA6b plays a pivotal role in pant responses to abscisic acid and in thermotolerance (Huang *et al.*, 2016) as well as ROS accumulation and the expression of antioxidant genes (Wenjing *et al.*, 2020). Other transcripts that were increased in abundance include DRM2, which is important in plant defence responses (Roy *et al.*, 2020), and KMD4, which targets type-B ARR proteins for degradation and is required for cytokinin responses through control of transcription factors (Kim *et al.*, 2013).

Transcripts encoding enzymes and proteins involved in plant responses to hypoxia, such as unknown proteins 26 and 32, were increased in roots exposed to *P. oryzihabitans* (Fig. 5B). Severe oxygen depletion can suppress LR formation (Shukla *et al.*, 2019; Pedersen *et al.*, 2021). The uptake of oxygen in respiration by the bacteria may contribute to some of the observed metabolic adaptations in the transcriptome signature (Pucciariello and Perata, 2021). Other genes that were highly expressed in the presence of bacteria encode proteins that are involved in nutrient acquisition and transport. For example, the levels of transcripts encoding several root hair-specific proteins including RHS7, RH17, and RH18, and a number of transporters such as the sucrose transporter SUC2, the POLYOL/monosaccharide transporter PMT6, the boron transporter 1 BOR1, the aluminium-activated malate transporter ALMT1, and the zinc transporter 3 precursor ZIP3 were higher in roots in the presence of *P. oryzihabitans.* Similarly, the levels of transcripts encoding HAK5 that is required for plant growth and K^+^ acquisition particularly under saline conditions (Nieves-Cordones *et al.*, 2010) were significantly higher in the roots exposed to *P. oryzihabitans*, as were transcripts encoding the MYB transcription factor MYBL2, which is a key negative regulator of anthocyanin biosynthesis in response to changes in sucrose availability (Dubos *et al.*, 2008).

The expression of genes encoding UDP-glycosyltransferases UGT91A1, UGT78D4, UGT84A1, and UGT78D1, as well as those encoding transparent testa TT7 and GST26, which play an important role in regulating the availability of secondary metabolites, was lower in bacteria-exposed roots. Similarly, transcripts encoding GA3OX, which catalyses the conversion of precursor GAs to their bioactive forms during vegetative growth (Mitchum *et al.* 2006), were significantly lower in the roots exposed to *P. oryzihabitans*.

Targeted ROS production is crucial to the hormone-dependent regulation of RSA (Eljebbawi *et al.*, 2021). For example, hyrdogen peroxide is required for brassinosteroid-mediated cell division in the root quiescent centre and for seedling development (Tian *et al.*, 2018). The data presented here provide evidence that ROS signalling is important in RSA responses to *P. oryzihabitans.* For example, while levels of ERF109 transcripts were not changed in the roots exposed to bacteria, the *P. oryzihabitans*-induced changes in RSA were absent from the *erf109* mutants. ERF109 is involved in the amplification of ROS signalling and systemic transmission of ROS signals in response to biotic and abiotic stresses (Bahieldin *et al.*, 2016), as well as in the JA-dependent regulation of RSA (Xu *et al.*, 2020).

The *P. oryzihabitans-*induced changes in RSA were similar in the *vtc* mutants that are deficient in the low molecular weight antioxidant ascorbate (Foyer *et al.* 2020) and the WT plants. This finding demonstrates that changes in total antioxidant capacity alone are not important in plant–bacteria interaction. The *vtc* mutants have modified phytohormone signalling pathways (Kerchev *et al.*, 2013; Caviglia *et al.*, 2018) but these changes do not influence the responses of RSA to *P. oryzihabitans.* In contrast, the *P. oryzihabitans-*induced changes in LR density were absent from the *cad2-1*, *pad2-1*, and *rax1-1* mutants, indicating that GSH-mediated redox regulation is important in root responses to the bacterium. GSH is essential for root development (Passaia *et al.*, 2014, Ehrary *et al.*, 2020). The GSH-deficient *rootmeristemless1* (*rml1*) mutant is unable to develop roots because of impaired root apical meristem functions (Vernoux *et al.*, 2000). The glutathione reductase-deficient *miao* mutants also show poor root growth (Yu *et al.*, 2013). Mutants lacking glutathione peroxidases have modified root phenotypes (Passaia *et al.*, 2014). Crucially, glutaredoxins (GRXs) such as GRXS8 and GRXS17 are involved in the regulation of RSA (Ehrary *et al.*, 2020; Martins *et al.*, 2020). GSH enhances the sensitivity of roots to auxin (Pasternak *et al.*, 2020) and is required for the conversion of indole butyric acid (IBA) to IAA (Trujillo-Hernandez *et al.*, 2020). The data presented here demonstrate that the root GSH pool is essential for the facilitation of bacteria-driven changes in RSA.

The GSH pool is involved in the SL-dependent control of RSA through the MAX2 protein (Marquez-Garcia *et al.*, 2014). SLs are important in rhizosphere communication (Bouwmeester *et al.*, 2007) and are required for plant responses to nutrient deficiencies (Shindo *et al.*, 2020). They are required for the initiation of symbiotic interactions with arbuscular mycorrhizal fungi, when nutrients are limiting (Akiyama *et al.*, 2005; Aliche *et al.*, 2020). The bacteria-induced increases in LR density were absent from mutants that are defective in SL synthesis or signalling, demonstrating the essential role of these phytohormones in plant–bacteria interactions.

In summary, evidence is presented showing that the root system of *A. thaliana* seedlings is changed in the presence of *P. oryzihabitans* PGP01 in a manner that suggests that this bacterium functions as a PGPR. Moreover, the observed changes in the root transcript profile are due to increases in mRNAs encoding proteins involved in mineral nutrition and phytohormone signalling but not defence or immune responses. Crucially, the data show that the long-distance perception of *P. oryzihabitans* PGP01 is sufficient to modulate RSA. ERF109, SLs, and GSH are key components required for the bacteria-mediated control of RSA. These findings demonstrate that SL and redox signalling are important factors in root responses to *P. oryzihabitans*, but changes in antioxidant capacity alone do not influence this process.

## Supplementary data

The following supplementary data are available at *JXB* online.

Fig. S1. Representative images of wild-type Arabidopsis seedlings that were separated by a 1 cm gap in the agar, by a 1 cm gap from seedlings growing in the presence of *P. oryzihabitans*, or separated from agar on which *P. oryzihabitans* was grown.

Table S1. Bacteria-induced changes in differentially expressed genes in *Arabidopsis thaliana* roots.

erac346_suppl_Supplementary_Figure_S1_Table_S1

## Data Availability

All RNAseq data from this article are available at the BioProject database (www.ncbi.nlm.nih.gov/bioproject/868724) under the accession number PRJNA868724.
