# Supplementary material for: Non-volatile signals and redox mechanisms are required for the responses of Arabidopsis roots to Pseudomonas oryzihabitans
Source: J Exp Bot. 2022 Aug 24;73(19):6971–82. doi: 10.1093/jxb/erac346 (PMC10277831; doi:10.1093/jxb/erac346)
Supplement: erac346_suppl_Supplementary_Figure_S1_Table_S1 [file erac346_suppl_supplementary_figure_s1_table_s1.pdf]

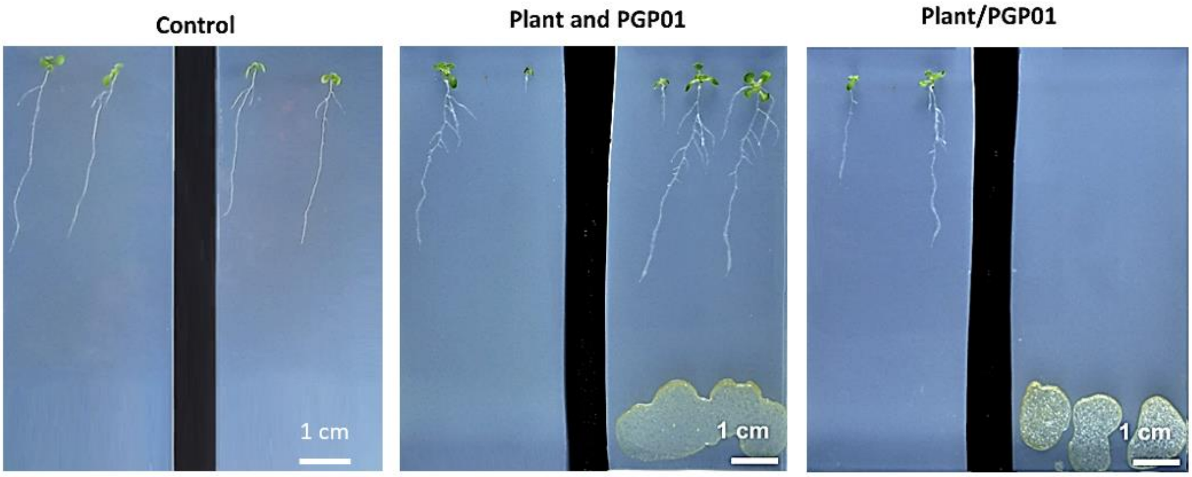

Supplemental Figure 1. Representative images of wild type Arabidopsis seedlings that were separated by a 1 cm gap in the agar (Control) or separated by a 1 cm gap from seedlings growing in the presence of *P. oryzihabitans* (Plants and PGP01), or separated from agar on which *P. oryzihabitans* was grown (Plants/PGP01). Seedlings were grown for 6 days in the absence of *P. oryzihabitans* and then for a further 7 days either in the absence of bacteria or in the presence of bacteria.

Supplemental Table 1. Bacteria-induced changes in differentially expressed genes in *Arabidopsis thaliana* roots. Seedlings had been grown for 6 days in the absence of *P. oryzihabitans* and then for a further 7 days either in the absence or presence of bacteria.

Gene no.	Annotation	log2FC	*p* value	padj
AT5G45950	GGL28, GDSL-motif esterase/acyltransferase/lipase	5.6279	2.68891154960748e-05	0.00139221084392477
AT3G22830	HSFA6B, heat shock transcription factor 6B	2.7659	2.22425506138743e-05	0.00119298718983145
AT4G13420	HAK5, potassium channel transporter	2.6987	0.000131440807244487	0.00514527159971563
AT2G39980	HXXXD-type acyl-transferase family protein	2.5914	2.16236341389664e-15	1.04961120110543e-12
AT5G28610	ATP-dependent RNA helicase DRS1-like protein	2.4686	3.37694115776828e-05	0.00168119716715971
AT3G07105	Nat-RNA	2.4621	0.000100518910936512	0.00413667663429937
AT5G47220	ATERF2, ETHYLENE RESPONSIVE ELEMENT BINDING FACTOR 2	2.3889	6.10117002245741e-05	0.00271697975128516
AT1G71030	MYBL2, MYB family transcription factor L2	2.3310	1.37372491912508e-07	1.4339915607383e-05
AT1G19530	RGAT1, RGA TARGET 1	2.2366	5.82043863853518e-21	4.9134624611217e-18
AT4G28850	XTH26, XYLOGLUCAN ENDOTRANSGLUCOSYLASE/HYDROLASE	2.2301	4.11106823558365e-15	1.9004881157641e-12
AT1G23052	other RNA	2.2079	0.000196217977034907	0.00708135360986945
AT2G42250	CYP712A1, CYTOCHROME P450,	2.1592	1.80096045143351e-14	7.60161915761589e-12
AT3G26740	CCL, CCR-LIKE	2.1305	1.43122818419903e-07	1.48602815103788e-05
AT2G33830	ATDRM2, DORMANCY/auxin ASSOCIATED GENE 2,	2.1169	2.41560561157097e-43	4.69013985542619e-39
AT4G06746	DREB AND EAR MOTIF PROTEIN 5, RAP2.9	2.0956	2.64928161113032e-09	4.14826223884728e-07
AT1G69490	NAC transcription factor	2.0694	0.00025088435792351	0.00848636009310603
AT5G02020	SALT INDUCED SERINE RICH, SIS	2.0457	4.40986204975387e-17	2.761996179291e-14
AT1G76410	RING/U-box protein	1.9623	3.82211084225536e-10	7.57245960339083e-08
AT2G15880	LEUCINE-RICH REPEAT/EXTENSIN 10, LRX10	1.9556	2.21083308388765e-08	2.86170234378417e-06
AT1G52820	2OG oxygenase	1.9237	3.33662705445412e-19	2.69933128705338e-16
AT1G54970	RHS7, ROOT HAIR SPECIFIC 7	1.9116	2.441416408325e-27	1.18506352460096e-23
AT4G36670	ATPMT6, PLT6, PMT6, POLYOL TRANSPORTER 6,	1.8883	2.64143954854749e-05	0.00137678389953607
AT1G18100	E12A11, MFT, MOTHER OF FT AND TFL1	1.8304	1.60619904299901e-07	1.64136634836151e-05
AT2G34180	CIPK13, SNF1-RELATED PROTEIN KINASE 3.7, SNRK3.7	1.8210	1.32921302660277e-07	1.3950270337578e-05

AT3G15450	Aluminium induced protein with YGL and LRDR motif	1.8116	8.85204506895031e-06	0.000549109607216419
AT5G17860	CALCIUM EXCHANGER 7, CAX7, CCX1	1.7898	9.59847694705214e-06	0.000584213255184841
AT2G42850	CYP718, CYTOCHROME P450, FAMILY 718	1.7749	1.02605821845978e-15	5.10819137682437e-13
AT5G53980	ATHB52, HB52, HOMEOBOX PROTEIN 52	1.7665	3.928179209114e-11	1.05929899339107e-08
AT3G23880	F-box and associated interaction domains-containing protein	1.7574	7.12593271224363e-06	0.000455122070858297
AT3G23150	ETHYLENE RESPONSE 2, ETR2	1.7524	2.20233558352142e-17	1.42535158965506e-14
AT5G54585	hypothetical protein	1.7496	1.18794038396234e-06	9.64896248421601e-05
AT1G52342	protein-encoding	1.7074	9.44409902014703e-22	1.13232006181823e-18
AT1G21400	THDP-binding	1.6833	1.64004438962751e-11	4.82471240439511e-09
AT2G15890	CBP1, CCG-BINDING PROTEIN 1	1.6800	9.91421562160583e-22	1.13232006181823e-18
AT5G06570	alpha/beta-Hydrolases superfamily protein	1.6779	5.79812925513363e-22	7.50509850784497e-19
AT1G33055	HUP32, HYPOXIA RESPONSE UNKNOWN PROTEIN 32	1.6547	1.03954541868515e-10	2.46144071331595e-08
AT5G24490	30S ribosomal protein	1.6480	1.02809146489359e-17	6.88324961461168e-15
AT5G57540	XTH13, XYLOGLUCAN ENDOTRANSGLUCOSYLASE/HYDROLASE	1.6402	1.64278100156001e-10	3.54402621403212e-08
AT5G65207	HP-response to hypoxia	1.6301	1.17532762737464e-22	1.75539701639277e-19
AT2G22980	SCPL13	1.6233	2.89104501825565e-05	0.00147717184406452
AT1G80380	GLYCERATE KINASE, GLYK, photorespiration	1.5966	0.000100561978336903	0.00413667663429937
AT1G11185	FASCICLIN-LIKE ARABINOGALACTAN 6, FLA6	1.5822	4.92502093882285e-11	1.25821324405506e-08
AT3G48360	BTB/POZ and TAZ domain-containing protein 2	1.5805	2.63236720081918e-05	0.00137678389953607
AT4G15990	Hypothetical protein	1.5721	1.72207258632121e-06	0.0001306084427188
AT3G10020	HUP26, HYPOXIA RESPONSE UNKNOWN PROTEIN 26	1.5665	2.3034525991779e-23	3.72698630546984e-20
AT1G76590	PLATZ transcription factor family protein	1.5514	1.78002796122821e-10	3.7979146038689e-08
AT1G80440	KMD, kiss me deadly	1.5265	3.27624729760349e-15	1.55150286659193e-12
AT2G31083	CLAVATA3/ESR (CLE)-related protein 5	1.5115	0.000226899958861721	0.00790931705791593
AT4G26288	uncharacterised protein	1.4865	2.23459415907147e-06	0.000163123664301977
AT5G57530	XTH12, xyloglucan endotransglucosylase/hydrolase 12	1.4792	2.06393347707639e-13	7.56100611149344e-11
AT2G39400	Alpha/beta-Hydrolases superfamily protein	1.4691	1.81021383458098e-07	1.81170679444455e-05
AT1G09932	Phosphoglycerate mutase family protein	1.4647	0.000220814119634741	0.0077528516217507
AT5G47240	NUD8, Nudix hydrolase 8	1.4558	2.50392129621379e-08	3.19842999258467e-06
AT5G49450	BZIP1, Basic leucine zipper 1	1.4274	4.8821201267715e-16	2.63309012170543e-13
AT5G61590	Ethylene-responsive transcription factor ERF107	1.4254	5.01600337274746e-17	3.04346004641452e-14
AT4G12470	AZI1, pEARLI1-like lipid transfer protein 1	1.4232	4.49250842163152e-11	1.17873707451889e-08
AT5G56550	OXS3, OXIDATIVE STRESS 3	1.4037	5.15938180633027e-08	6.07118528192172e-06
AT5G56100	Glycine-rich protein / oleosin, MDA7.16	1.3796	2.46219479272488e-06	0.000177059163316838
AT2G20520	FLA6, Fasciclin-like arabinogalactan protein 6	1.3723	1.99256254897142e-09	3.30663200434437e-07
AT2G23030	SNF1-RELATED PROTEIN KINASE 2.9, SNRK2-9	1.3664	3.85703540762127e-08	4.68051246714841e-06
AT4G05070	WIP2, WOUND-INDUCED POLYPEPTIDE 2	1.3552	5.5342675799837e-18	3.97975330862828e-15
AT1G70290	trehalose-6-phosphate synthase (TPS)-like	1.3438	8.52234868879961e-15	3.6771093809274e-12
AT3G59480	FRK4, FRK7, FRUCTOKINASE 4, FRUCTOKINASE 7	1.3374	2.17583505247653e-05	0.00117024967808544
AT1G01453	ATMLKL1, MIXED LINEAGE KINASE LIKE 1 cell death regulation	1.3300	0.000102634361749067	0.00419526056362081
AT1G10140	Uncharacterized conserved protein UCP031279	1.3279	1.75336798694292e-18	1.36173571337935e-15
AT3G59940	KFB50, KELCH REPEAT F-BOX 50 negative cytokinin response	1.3251	2.60112511027128e-09	4.10597114967701e-07
AT1G75030	PR5-like protein, thaumatin, TLP3	1.3192	1.19270240843214e-06	9.64896248421601e-05
AT4G34530	Transcription factor bHLH63	1.3070	0.000209683194352143	0.00745642655959927
AT5G39580	PER62, peroxidase cell wall-targeted protein	1.3000	2.07622632082536e-07	2.06728257667412e-05
AT2G25900	Zinc finger CCCH domain-containing protein 23	1.2817	3.73523862432998e-13	1.29506059160698e-10
AT2G40000	HSPRO2 nematode resistance protein	1.2588	2.75246288779175e-13	9.89663322766013e-11
AT3G49960	PER35, peroxidase 35	1.2578	3.88959996164479e-14	1.57334318448532e-11
AT1G08430	ALMT1, ALUMINUM-ACTIVATED MALATE TRANSPORTER 1	1.2425	5.91720744861379e-16	3.1050945897915e-13
AT1G64660	ATMGL, METHIONINE GAMMA-LYASE, MGL	1.2357	3.2947912045728e-10	6.7338595818932e-08
AT1G03090	MCCA I leucine degradation	1.2325	1.74890001900902e-11	5.06815563717599e-09
AT2G44080	ARGOS-LIKE, ARL	1.2223	1.36397726814292e-09	2.34362678214717e-07
AT1G21130	IGMT4 Indole glucosinolate O-methyltransferase 4	1.2176	4.90270404275955e-05	0.00228275543631222

AT5G52790	DUF21 domain-containing protein At5g52790	1.2093	3.91830591086601e-10	7.65323135621687e-08
AT4G39780	Ethylene-responsive transcription factor ERF060	1.2030	1.15237654524528e-10	2.69572807258824e-08
AT4G38470	Serine/threonine-protein kinase STY46	1.1720	9.4593080585681e-05	0.00394124303144116
AT3G16150	Probable isoaspartyl peptidase/L-asparaginase 2	1.1713	0.000112783260795733	0.00452371951926209
AT2G47160	BOR1 boron transporter 1	1.1703	1.18703171604154e-16	6.58497365676071e-14
AT4G40070	RING-H2 finger protein ATL32	1.1545	7.53816739881444e-07	6.44762370992869e-05
AT4G27450	AILP1, aluminum induced protein, response to hypoxia	1.1529	6.74557443664398e-10	1.21270438205444e-07
AT1G34510	PER8, peroxidase	1.1507	2.74701435044448e-06	0.000194657046088431
AT1G26800	MPSR1, E3 ligase	1.1497	2.50824398326016e-05	0.00132697725283322
AT5G66650	CMCU, Calcium uniporter protein 3	1.1274	0.000103319269556027	0.00421438432289878
AT4G24230	ACBP3, Acyl-CoA-binding domain protein	1.1254	2.3002220243354e-07	2.25561165780283e-05
AT3G52840	BGAL2, Beta-galactosidase 2	1.1242	1.94790127861457e-06	0.000144905943393029
AT5G18170	GDH1; glutamate dehydrogenase 1	1.1236	6.38203786751723e-11	1.58863650302198e-08
AT2G13360	AGT1, alanine:glyoxylate aminotransferase	1.1192	2.26322469845033e-08	2.91011726788819e-06
AT5G22410	RHS18 root hair specific 18, root elongation branching	1.1150	3.94171371869431e-10	7.65323135621687e-08
AT2G02710	PAS/LOV protein B	1.1096	3.5381564054876e-11	9.67561193928835e-09
AT2G18700	TPS11 trehalose phosphatase/synthase 11	1.1068	5.54263054404168e-08	6.48287437609116e-06
AT1G48690	Auxin-responsive GH3 family protein	1.0992	2.63309720503774e-08	3.31975424240342e-06
AT4G38390	RHS17, root hair specific 17	1.0959	3.94979583030177e-05	0.00191755904214962
AT5G07010	ST2A sulfotransferase 2A; response to JA	1.0900	1.57714325629434e-06	0.000120558320725239
AT1G52343	transmembrane protein	1.0862	0.00030435674169928	0.00983259650055445
AT4G39675	hypothetical protein	1.0832	3.77473459432065e-05	0.0018414634895309
AT4G08290	nodulin MtN21-like transporter family protein	1.0825	1.35071465547663e-10	3.04947392450399e-08
AT3G45300	IVD, isovaleryl-CoA-dehydrogenase	1.0813	8.98657844966844e-14	3.42124327801495e-11
AT1G23870	TPS9-like trehalose-phosphatase/synthase 9	1.0661	5.96105475438925e-11	1.50311479365223e-08
AT5G63270	RPM1-interacting protein 4 (RIN4)	1.0650	2.0252466656941e-06	0.000150084691836323
AT4G07960	xyloglucan glycosyltransferase 12, CSLC12	1.0557	1.63133029389859e-05	0.000923437579776532
AT1G56220	DRMH3 Dormancy/auxin associated family protein	1.0468	3.6088762670339e-21	3.68789166319632e-18
AT5G56870	beta-galactosidase 4	1.0449	2.56746982259021e-18	1.91730746443891e-15
AT5G14120	Major facilitator superfamily protein	1.0437	1.32253215275375e-13	4.93813159189745e-11
AT3G15630	uncharacterised protein	1.0421	2.00275334246087e-14	8.27350189302558e-12
AT4G23410	TET5, TETRASPANIN5	1.0339	0.000138469672662713	0.00538782998881611
AT5G01210	HXXXD-type acyl-transferase family protein	1.0328	6.34197542900807e-10	1.15080182177216e-07
AT1G70885	pseudogene of MLP-like protein 31	1.0299	0.000230162026976638	0.00796582159675296
AT1G22710	SUCROSE-PROTON SYMPORTER 2,	1.0258	2.20090132109901e-07	2.16917259139383e-05
AT2G32150	Haloacid dehalogenase-like hydrolase superfamily protein	1.0254	6.03413431094412e-14	2.39099493431206e-11
AT1G77885	hypothetical protein	1.0245	1.32360357477081e-07	1.3950270337578e-05
AT4G30270	XTH, SEN4, SENESCENCE 4	1.0238	1.70228399204697e-08	2.26380451983452e-06
AT4G02520	GLUTATHIONE S-TRANSFERASE PHI 2, GST2, GSTF2	1.0223	2.04716213732967e-11	5.8452500085872e-09
AT4G29905	unknown protein	1.0212	6.016618927541e-18	4.17209546775486e-15
AT4G29190	OZF2, OXIDATION-RELATED ZINC FINGER 2	1.0179	0.000101948935706689	0.0041760348854031
AT5G25110	CIPK25, SnRK3.25	1.0120	3.50908287636272e-05	0.00172486969942933
AT3G01290	HIR2, HYPERSENSITIVE INDUCED REACTION 2	0.9960	9.06954392996608e-17	5.17924308659475e-14
AT1G43160	ethylene response factor RAP2.6	0.9934	1.34021973775618e-12	4.26585351283181e-10
AT5G48175	transmembrane protein	0.9923	7.30205616101363e-05	0.00318086175550199
AT1G73655	FKBP-like peptidyl-prolyl cis-trans isomerase	0.9920	9.37445304440774e-05	0.0039142877486069
AT2G19810	OXIDATION-RELATED ZINC FINGER 1	0.9968	0.000183511196015615	0.00671008169837889
AT4G35750	SEC14 cytosolic factor family protein	0.9952	4.11684828852192e-21	3.99663631849708e-18
AT5G21940	signal transduction histidine kinase M-like protein	0.9946	2.74378195630332e-08	3.43698519119905e-06
AT5G14780	FDH, NAD-dependent formate dehydrogenase	0.9986	1.31209558250076e-10	3.03281521783748e-08
AT2G32270	ZINC TRANSPORTER 3 PRECURSOR, ZIP3	0.9974	7.6188335319868e-08	8.6507176524594e-06
AT1G63180	UDP-D-GLUCOSE/UDP-D-GALACTOSE 4-EPIMERASE 3, UGE3	0.9972	5.87286882460343e-06	0.000383931384170034
AT1G61740	Sulfite exporter TauE/SafE family protein	0.9936	5.6444978569045e-10	1.03942719657149e-07

AT4G25790	CAPE4 (Cysteine-rich secretory protein	0.9986	2.09337592244479e-05	0.00114171311545472
AT1G23850	transmembrane protein	0.9963	2.13228542115338e-05	0.00115321598153521
AT5G59780	MYB DOMAIN PROTEIN 59, MYB59	0.9936	3.53053247177683e-10	7.14050192416863e-08
AT3G47160	RING/U-box superfamily protein	0.9925	5.52709991134395e-10	1.03186703729475e-07
AT1G23760	JP630, PG3, PGL1, POLYGALACTURONASE 3	0.9922	0.000139017885962155	0.00539834254768241
AT1G19400	E-4PDHASE, ERYTHRONATE-4PHOSPHATE DEHYDROGENASE	0.9916	9.22607473978682e-08	1.01780379061194e-05
AT5G13080	WRKY DNA-BINDING PROTEIN 75, ATWRKY75	0.9915	2.5398055704222e-08	3.22306306897499e-06
AT5G48570	ROF2, (CC)-tetratricopeptide repeat protein	0.9928	3.43595892683817e-05	0.00170185149294617
AT3G23810	S-ADENOSYL-L-HOMOCYSTEINE (SAH) HYDROLASE 2, SAHH2	-0.9960	1.070185544797e-12	3.46312042296309e-10
AT4G27030	FAD4, FADA, FATTY ACID DESATURASE 4	-0.9943	3.07385524605985e-06	0.000213913883360208
AT3G23470	Cyclopropane-fatty-acyl-phospholipid synthase	-0.9920	2.09016484966872e-09	3.41030594295529e-07
AT5G02490	HSP70-2, heat shock 70-2	-0.9913	9.31344921336762e-06	0.000574063269608717
AT5G13930	ATCHS, CHALCONE SYNTHASE	-0.9991	0.00001385676788000	2.14633951727439e-07
AT5G24120	ATSIG5, SIGMA FACTOR 5	-1.0031	1.23071587386647e-07	1.31294392346107e-05
AT4G17460	HAT1 HD-ZIP protein that regulates meristematic activity	-1.0155	2.81932499328916e-10	5.823405752096e-08
AT1G32380	PHOSPHORIBOSYL PYROPHOSPHATE (PRPP) SYNTHASE 2, PRS2	-1.0309	3.95075783916137e-09	5.99280579727791e-07
AT1G60270	BETA GLUCOSIDASE 6, BGLU6	-1.0420	3.51301728717702e-06	0.000240171632562778
AT5G56080	NAS2, NICOTIANAMINE SYNTHASE 2	-1.0551	5.09147545433218e-10	9.5976783904188e-08
AT3G07720	Galactose oxidase/Kelch repeat superfamily protein	-1.0595	7.03487827106991e-13	2.31507112730667e-10
AT4G28660	PHOTOSYSTEM II REACTION CENTER PSB28 PROTEIN, PSB28	-1.0655	0.000286105037258149	0.00933616034185582
AT3G23530	Cyclopropane-fatty-acyl synthase	-1.0683	9.2008379526565e-11	2.20547493442936e-08
AT4G25700	BCH1, BETA CAROTENOID HYDROXYLASE	-1.0689	0.000252382721461291	0.0085221963824216
AT3G18000	N-METHYLTRANSFERASE 1, NMT1	-1.0737	1.01100742129387e-08	1.44336177145895e-06
AT4G37400	CYP81F3, CYTOCHROME P450	-1.0770	1.41297040255126e-06	0.000110177643919418
AT1G19850	ARF5, AUXIN RESPONSE FACTOR 5	-1.0814	3.50961165188095e-06	0.000240171632562778
AT5G58770	Cis-PRENYLTRANSFERASE 4	-1.1025	5.0012760908425e-05	0.00231201848999519
AT5G64510	TIN1, TUNICAMYCIN INDUCED 1	-1.1088	4.85604017317235e-05	0.00227192472294733
AT4G37370	CYP81D8, CYTOCHROME P450	-1.1223	0.000198794920653553	0.00716104300447009
AT5G08050	RIQ1 a grana core localized protein	-1.1331	2.15598360454525e-07	2.13574375846176e-05
AT4G01630	EXPANSIN 17	-1.1427	4.29287760827457e-06	0.00028841007488671
AT4G25640	DETOXIFYING EFFLUX CARRIER 35	-1.1428	6.57664849095124e-24	1.27692207100309e-20
AT5G67370	CGLD27, CONSERVED IN GREEN LINEAGE AND DIATOMS 27	-1.1518	3.96652548073149e-05	0.00192055009311428
AT1G19050	RESPONSE REGULATOR 7	-1.1528	2.64456424398055e-07	2.50472484688421e-05
AT2G16890	UDP-glycosyltransferase protein	-1.1641	2.23480092214287e-06	0.000163123664301977
AT3G06325	Nat-RNA	-1.1667	4.01000785556634e-05	0.00193677394337503
AT1G10960	ATFD1, FD1, FERREDOXIN 1	-1.1911	4.66056335721969e-15	2.10440693357622e-12
AT3G41761	other RNA	-1.1921	0.000242156657741818	0.00827766490618863
AT5G17050	UDP-GLUCOSYL TRANSFERASE 78D2, UGT78D2	-1.1939	8.48014263990729e-14	3.2930089899288e-11
AT2G04965	small_nucleolar_RNA	-1.2267	9.80269500302612e-05	0.00405819032364083
AT1G05650	Pectin lyase-like superfamily protein	-1.2383	9.48002320986851e-06	0.000582481426084832
AT2G46420	helicase with zinc finger protein	-1.2450	2.33961372719593e-07	2.27381787617491e-05
AT2G01008	maternal effect embryo arrest protein	-1.2568	1.26688522695938e-06	0.000101314365882974
AT4G10310	HKT1, HIGH-AFFINITY K+ TRANSPORTER 1	-1.2584	2.2408784885406e-05	0.00119859219651527
AT1G78570	RHAMNOSE BIOSYNTHESIS 1	-1.2604	1.06158911941489e-25	2.57647679281994e-22
AT3G12820	MYB transcription factor	-1.2974	1.73659034360266e-06	0.000131197035452876
AT1G10370	EARLY-RESPONSIVE TO DEHYDRATION 9,	-1.3056	2.13583030857966e-11	6.01004076396852e-09
AT5G04950	NAS1, NICOTIANAMINE SYNTHASE 1	-1.3249	7.13266857685643e-05	0.00312613754149536
AT3G47420	Encodes protein AtPS3.	-1.3363	2.11561189504548e-10	4.46486092980468e-08
AT4G04840	METHIONINE SULFOXIDE REDUCTASE B6	-1.3391	4.70251545512974e-21	4.1501836398545e-18
AT5G48880	peroxisomal 3-keto-acyl-CoA thiolase 2	-1.3454	3.96843906634643e-13	1.35177566512601e-10
AT1G71850	Ubiquitin carboxyl-terminal hydrolase	-1.3483	0.000110186836054644	0.00444779128656334
AT2G03855	microRNA	-1.3993	3.16029455343753e-05	0.00158745722404901
AT3G21560	UDP-GLUCOSYL TRANSFERASE 84A2, U	-1.4643	3.53200926758001e-13	1.24686348980606e-10

AT4G19690	IRON-REGULATED TRANSPORTER 1, IRT1	-1.4696	9.33445406113193e-05	0.00390598620799434
AT1G06000	flavonol-7-O-rhamnosyltransferase	-1.4989	9.46323087085715e-27	3.67476181177125e-23
AT4G11050	glycosyl hydrolase 9C3	-1.5065	0.000119262005512482	0.00474506372752119
AT5G62420	NAD(P)-linked oxidoreductase superfamily	-1.5687	2.30293464361313e-05	0.00122503504220253
AT1G73120	F-box/RNI superfamily protein	-1.5884	0.000210580676296159	0.00747465157397847
AT1G01580	FRO2, low-iron-inducible ferric chelate	-1.5939	0.000108201752611506	0.00437676089313543
AT4G14690	EARLY LIGHT-INDUCIBLE PROTEIN 2, ELIP2	-1.6223	1.34331312703883e-06	0.000106892490469615
AT5G05270	ATCHIL, CHALCONE ISOMERASE LIKE, CHIL	-1.6382	1.15430849386302e-23	2.03745942880403e-20
AT5G02780	GLUTATHIONE TRANSFERASE LAMBDA 1,	-1.6994	1.68473669628636e-05	0.000945556624772448
AT3G55120	CHALCONE FLAVANONE ISOMERASE, TT5	-1.6996	5.25911584361047e-26	1.45872847456487e-22
AT5G16530	PIN5, PIN-FORMED 5	-1.7499	2.04173381800419e-05	0.00112301143938723
AT3G51240	F3H, FLAVANONE 3-HYDROXYLASE	-1.7759	3.67027526269819e-33	3.5631032250274e-29
AT5G62210	Embryo-specific protein 3	-1.8187	9.39330756216234e-10	1.67321522593527e-07
AT4G17680	EBS1, EXCLUSIVELY SENSITIVE TO BICARBONATE 1	-1.9719	9.26799461400036e-07	7.62488912819623e-05
AT1G30530	UDP-GLUCOSYL TRANSFERASE 78D1	-1.9790	2.50333971542691e-12	7.7150545896395e-10
AT1G80340	gibberellin 3 β-hydroxylase, GA3OX2	-2.1537	1.41356359700085e-09	2.40752199994461e-07
AT5G08640	FLAVONOL SYNTHASE 1, FLS1	-2.1546	7.20377836792845e-31	4.66228535972329e-27
AT3G12900	S8H, SCOPOLETIN 8- HYDROXYLASE	-2.1581	3.47043080260418e-05	0.00171455176751559
AT2G23910	NAD(P) binding Rossmann protein	-2.2752	2.23613393239031e-25	4.82408627014336e-22
AT3G22840	EARLY LIGHT-INDUCABLE PROTEIN, ELIP1	-2.5339	2.95149455693292e-07	2.78185525812667e-05
AT1G65060	4-coumarate: CoA LIGASE 3, 4CL3	-2.5341	2.39472422716175e-26	7.74932759909543e-23
AT5G17220	GLUTATHIONE S-TRANSFERASE PHI 12	-2.5665	7.68868622223284e-06	0.000483118225536805
AT5G07990	CYP75B1 TRANSPARENT TESTA 7, TT7	-2.7015	3.27567717962573e-22	4.54289629425808e-19
AT4G15480	UDP-Glycosyltransferase, UGT84A1	-2.8570	9.06769185751218e-17	5.17924308659475e-14
AT5G17040	UDP-Glycosyltransferase, UGT78D4	-3.1817	1.07533533475919e-11	3.21210936302837e-09
AT2G22590	UDP-Glycosyltransferase, UGT84A1	-3.9463	4.43121217421609e-21	4.09697217021807e-18
